# Nature–Based Interventions for Improving Health and Wellbeing: The Purpose, the People and the Outcomes

**DOI:** 10.3390/sports7060141

**Published:** 2019-06-10

**Authors:** Danielle F. Shanahan, Thomas Astell–Burt, Elizabeth A. Barber, Eric Brymer, Daniel T.C. Cox, Julie Dean, Michael Depledge, Richard A. Fuller, Terry Hartig, Katherine N. Irvine, Andy Jones, Heidy Kikillus, Rebecca Lovell, Richard Mitchell, Jari Niemelä, Mark Nieuwenhuijsen, Jules Pretty, Mardie Townsend, Yolanda van Heezik, Sara Warber, Kevin J. Gaston

**Affiliations:** 1 Zealandia Centre for People and Nature, 6012 Wellington, New Zealand; 2 Population Wellbeing and Environment Research Lab (PowerLab), School of Health and Society, University of Wollongong, 2522 Wollongong, Australia; thomasab@uow.edu.au; 3 School of Public Health, University of Queensland, Brisbane, 4006 Queensland, Australia; e.barber@uq.edu.au; 4 Discipline of Psychology, Australian College of Applied Psychology, Brisbane, 4000 Queensland, Australia; e.brymer@leedsbeckett.ac.uk; 5 Environment & Sustainability Institute, University of Exeter, Cornwall TR10 9EZ, UK; D.T.C.Cox@exeter.ac.uk (D.T.C.C.); K.J.Gaston@exeter.ac.uk (K.J.G.); 6 School of Public Health, University of Queensland, Brisbane, 4006 Queensland, Australia; j.dean@sph.uq.edu.au; 7 European Centre for Environment and Human Health, University of Exeter Medical School, Exeter EX1 2LU, UK; michael.depledge@pms.ac.uk; 8 School of Biological Sciences, University of Queensland, Brisbane, 4072 Queensland, Australia; r.fuller@uq.edu.au; 9 Institute for Housing and Urban Research, Uppsala University, 75120 Uppsala, Sweden; terry.hartig@ibf.uu.se; 10 Social, Economic and Geographical Sciences, James Hutton Institute, Aberdeen AB15 8QH, UK; katherine.irvine@hutton.ac.uk; 11 Norwich Medical School, University of East Anglia, Norwich, Norfolk NR15 1LT, UK; a.p.jones@uea.ac.uk; 12 Centre for Biodiversity and Restoration Ecology, Victoria University of Wellington, 6012 Wellington, New Zealand; Heidy.Kikillus@vuw.ac.nz; 13 European Centre for Environment and Human Health, University of Exeter Medical School, Truro TR1 3HD, UK; R.Lovell@exeter.ac.uk; 14 Centre for Research on Environment, Society and Health, University of Glasgow, Glasgow G12 8QQ, UK; richard.Mitchell@glasgow.ac.uk; 15 Department of Environmental Science, University of Helsinki, 00014 Helinski, Finland; jari.niemela@helsinki.fi; 16 ISGlobal, Barcelona Institute for Global Health, Barcelona Biomedical Research Park (PRBB), 08003 Barcelona, Spain; mnieuwenhuijsen@creal.cat; 17 Department of Biological Sciences, University of Essex, Colchester, Essex CO4 3SQ, UK; jpretty@essex.ac.uk; 18 School of Health & Social Development, Deakin University, 3217 Geelong, Australia; mardie.townsend@deakin.edu.au; 19 Zoology Department, University of Otago, 9016 Dunedin, New Zealand; yolanda.vanheezik@otago.ac.nz; 20 Integrative Medicine, The University of Michigan, Michigan, MA 48710, USA; swarber@med.umich.edu

**Keywords:** Nature–based health interventions, green prescriptions, wilderness therapy, forest schools, green exercise

## Abstract

Engagement with nature is an important part of many people’s lives, and the health and wellbeing benefits of nature–based activities are becoming increasingly recognised across disciplines from city planning to medicine. Despite this, urbanisation, challenges of modern life and environmental degradation are leading to a reduction in both the quantity and the quality of nature experiences. Nature–based health interventions (NBIs) can facilitate behavioural change through a somewhat structured promotion of nature–based experiences and, in doing so, promote improved physical, mental and social health and wellbeing. We conducted a Delphi expert elicitation process with 19 experts from seven countries (all named authors on this paper) to identify the different forms that such interventions take, the potential health outcomes and the target beneficiaries. In total, 27 NBIs were identified, aiming to prevent illness, promote wellbeing and treat specific physical, mental or social health and wellbeing conditions. These interventions were broadly categorized into those that change the environment in which people live, work, learn, recreate or heal (for example, the provision of gardens in hospitals or parks in cities) and those that change behaviour (for example, engaging people through organized programmes or other activities). We also noted the range of factors (such as socioeconomic variation) that will inevitably influence the extent to which these interventions succeed. We conclude with a call for research to identify the drivers influencing the effectiveness of NBIs in enhancing health and wellbeing.

## 1. Introduction

There are many pressing public health and environmental challenges associated with modern living, with rapidly growing levels of chronic, non–communicable physical and mental health conditions [1,2,3,4] and global recognition of serious health risks posed by stressful living conditions [5]. Engagement with nature is a common pursuit in cities [6] and it is becoming increasingly recognised as a means to alleviate many of these challenges. Evidence now points to benefits for physical health (e.g., lower prevalence of high blood pressure and allergies) [7,8], mental health (e.g., lower prevalence of depression and anxiety) [8,9,10,11] and social wellbeing outcomes [8] for people who spend time in nature. Moreover, there is evidence that the magnitude of such benefits can increase with the dose of nature [9]. It is thus of significant concern that urbanisation and the challenges of modern life are leading to reduced engagement with the natural environment [12]. 

To counter this development, nature–based health interventions (NBIs) can facilitate change through a somewhat structured promotion of nature–based experiences. NBIs are programmes, activities or strategies that aim to engage people in nature–based experiences with the specific goal of achieving improved health and wellbeing. For example, environmental manipulations where green and blue spaces are incorporated into cities can have positive outcomes associated with the management of habitats and flow of ecosystem services to people [13,14], but there is also a growing body of evidence highlighting the potential of green space for the treatment and prevention of physical, mental and social health and wellbeing challenges [8,15,16,17,18,19]. This recognition that experiences of nature can provide benefits for people represents a major shift in public health thinking for both the prevention and the treatment of health issues, beyond considering nature solely as a risk–factor (e.g., through the transmission of insect–borne diseases [20,21,22,23]).

Reflecting the growing body of research demonstrating a link between interactions with nature and health, many governments, non–government organisations, public and private stakeholders are now beginning to consider these potential benefits in their policy and planning frameworks [24,25,26,27]. Indeed, across the world, many NBIs are being implemented. These include, for example, minimum area targets for public green space [28] and ‘nature prescriptions’, where doctors or other health practitioners prescribe nature–based experiences for patients living with specific health conditions [29,30,31,32]. However, despite this growing movement, there is a dearth of guidance as to what NBIs are available and what specific health outcomes they might achieve and for whom. This can only limit the potential leveraging of natural settings to improve health and wellbeing outcomes for individuals and communities, potentially leading to inefficient and ill–targeted investment decisions. 

Here, we used expert elicitation to identify a range of NBIs that have been examined in the peer–reviewed scientific literature. This list of interventions is intended to provide a resource for decision–makers in government, non–government organisations, and other interested groups by outlining possible interventions, the potential health outcomes, and the target beneficiaries. 

## 2. Materials and Methods

**Figure 1:**
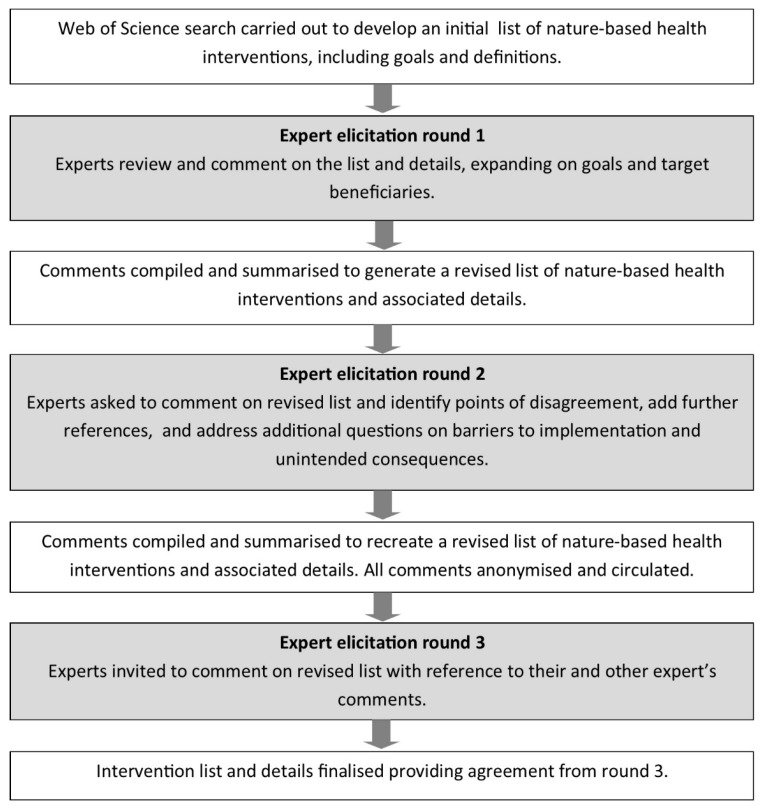
The Delphi expert elicitation process followed in this study. Tasks in boxes with no shading were carried out by D.F.S., those in shaded boxes involved all experts.

We used a Delphi expert elicitation process [33] to develop and then to refine and improve a list of NBIs that have received attention in the peer–reviewed scientific literature to date (Figure 1). The Delphi technique is an iterative method for building consensus. In this case, it was based on three rounds of questionnaires. Before the rounds of questions began, D.F.S. carried out a broad–reaching Web of Science literature search (initial search terms including ‘nature AND health OR wellbeing’, ‘nature–based health interventions’, ‘nature interventions’). The goal of this search was not a comprehensive review, but to develop a list of interventions—that is, programmes, activities or strategies that aim to engage people in nature experiences with the specific intention of improving health and wellbeing outcomes. The articles identified through the initial search were assessed, and NBIs identified where possible; further articles were found through the reference lists within the initial article set.

*Round 1.* In the first round, experts were asked to review and refine the list of interventions to ensure that those with similar methods but different names were removed. Experts were also invited to add intervention types and provide example references. Experts also commented on the definition, goals, and target beneficiaries of each intervention and identified further relevant literature. Thirty experts were invited to contribute. All are scientists and/or health practitioners actively publishing peer–reviewed research on the connection between people, nature and health and wellbeing. Nineteen participated. 

*Round 2.* Following the initial review process, the comments were compiled and summarised by D.F.S. This involved the revision of text to improve accuracy and incorporate new information from experts. This revised list was recirculated to all 19 experts, and they were invited to agree or disagree with the content. The experts were also provided with their own original comments during this step. At this point, the experts were also invited to answer further questions on four specific intervention types for which a significant body of literature was available and for which the panel of experts had specific expertise. The questions focused on the reach of the interventions, barriers to individuals and organisations in implementing the interventions and potential unintended negative consequences. 

*Round 3.* The intervention list was again revised by D.F.S. on the basis of all comments made, involving addition of detail and refinement of definitions and other text. Some experts provided significant in–depth detail that went beyond the scope of this study, and in these instances, the detail was summarised. All responses from round 2 were anonymised and recirculated to all 19 experts again to review their own answers on reflection of other expert’s answers and ensure that the revision conducted by D.F.S. accurately reflected their views and that a consensus had been reached. They were also invited to add final thoughts triggered by the comments that had been put forward by their peers.

All comments were synthesised to produce the final list presented in this article.

## 3. Results

Nineteen of 30 invited experts who were identified from across the world actively engaged with a Delphi expert elicitation process to review a compilation of NBIs identified through a literature search conducted by D.F.S. The 19 experts who participated in the review are all named authors on this paper. They represent a diversity of disciplines and areas of expertise relevant to the broad field of nature and health. Geographically, representation in the panel was particularly good from the United Kingdom and Oceania, while there were gaps in representation from Europe, Asia, Africa and the Americas. This was in part related to the availability of the identified experts to participate and in part, to difficulties in identifying experts who do not publish in English–language peer–review journals. The representation of national/cultural contexts in the literature reviewed, however, extended beyond those in which the 19 experts are situated. 

Twenty–seven distinct NBIs that have received some peer–reviewed research attention were summarised using the expert elicitation process (Table 1 and Table 2). Interventions were excluded from the list where health and wellbeing outcomes were not explicit goals (e.g., programmes that solely aimed to connect people with nature without the intention of also delivering health and wellbeing benefits). 

**Figure 2:**
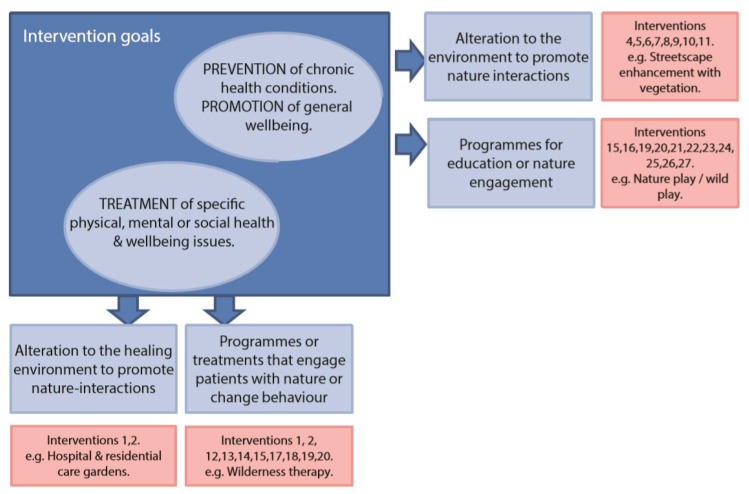
A categorisation schematic of the nature–based health interventions identified in an expert elicitation process. Numbers refer to interventions identified in Table 1 and Table 2.

The intended outcomes and target beneficiaries varied widely across interventions, from the promotion of wellbeing and the prevention of chronic or lifestyle–based health conditions (e.g., through the provision of public parks) to targeted treatments for people living with specific health conditions (e.g., nature prescriptions for reducing high blood pressure). A categorisation of the different interventions is given in Figure 2; some aim to *change the environment* in which people live (e.g., providing new or better quality public green spaces [18,28,34]; Table 1) and work (e.g., hospital, workplace), and others aim to *change people’s behaviour* and their interactions with nature (e.g., nature play/wild play programmes [35]; Table 2 and Figure 2). There was some overlap in these categories where people engaged in nature–based activities through interventions that also involved enhancing the environment (e.g., ‘green gyms’ or environmental volunteering; Table 2). 

A closer investigation of barriers and potential negative implications for four intervention types was carried out, specifically, green prescriptions, wilderness therapy, green gyms and outdoor exercise groups (Table 2). There were a number of commonalities in the barriers, which included knowledge of health practitioners and lack of access to the intervention (especially where it relied on having transport or could not be completed independently as it relied on a specific organised programme). There were also some potential unanticipated negative implications, with risks of physical injury a common theme.

## 4. Discussion

The scientific literature includes studies on a diverse suite of nature–based interventions through which ill health might be prevented, health and wellbeing can be promoted, and/or specific illnesses might be treated. These interventions could provide a useful tool for enabling and encouraging people to engage with nature and, in doing so, potentially receive a multitude of physical, mental and social health benefits. Broadly speaking, the interventions identified in this study can be grouped into actions that change the environment in which people live, work, learn, recreate or heal, and those that change people’s behaviour through programs or other means. Because of this, the scale of impact varies from the population to the individual level and in the level of effort needed to achieve outcomes [175]. Consequently, the selection of one intervention over another or the composition of a suite of interventions, must reflect the capacity and skills of the administering organisations, the goals of the activity or activities, as well as the needs of the population or the individual.

A key feature of nature–based health interventions is that a single intervention can affect people in multiple ways and, therefore, potentially improve wellbeing across a range of domains [15,17,176]. For example, nature prescriptions can both promote physical activity leading to many positive health outcomes, while also providing patients with the mentally restorative effects of natural spaces [32,98,99,177]. Thus, investment in interventions can achieve significant outcomes across multiple domains [17] and, when scaled up, could have significant and cost–effective implications for population health. Furthermore, nature can be pro–actively planned into city development activities to provide a protective factor against many health conditions [15,177]. Research into the extent and magnitude of these outcomes is critical to assist decision–makers (such as hospital or care–home managers and urban planners) in weighing up the costs and benefits of investing in the various options, identifying ways to coordinate efforts (e.g., with regard to the siting of health care facilities) and ultimately supporting ‘prevent–to–save’ initiatives [178].

As with other public health interventions, there are many factors that influence both the effectiveness and the success of NBIs. For example, the accessibility of public parks will inevitably influence their use by communities, and a number of studies have found people are more likely to exercise in neighborhoods with greater levels of park availability [11,59,179,180,181,182,183,184,185]. There are also social equity issues at play. For example, disadvantaged neighborhoods have been repeatedly found to have less vegetation cover, fewer public parks and fewer street trees; additionally, organised user–pays programmes may be inaccessible for some disadvantaged sectors of society [186,187,188,189]. Furthermore, the physical and mental capability of participants is a potential barrier to accessing some intervention types, as identified in this expert elicitation study. Social factors, such as acceptability of the intervention to local communities, are also likely to have an important influence on the uptake of nature–based health interventions; for example, several studies have now found that cultural differences have a critical influence on the use of public green spaces [190,191,192]. Finally, an individual’s age, gender and other factors will play a role, as will perceptions of nature and the appropriateness of the nature setting in its wider context (e.g., ecological characteristics of the nature setting, facilities and infrastructure, programmed activities and experiences of social inclusion in the setting) [193,194,195].

As NBIs are not yet mainstream within the health care community, practitioner buy–in and knowledge was identified as a particular challenge in this study. Further knowledge and communication about the effectiveness of interventions gained from rigorous research is therefore likely to be an important precursor for their use, including understanding the limitations or barriers to success and accounting for local contexts. Active evaluation and communication of findings from relevant studies is needed to build more solid foundations for decision–making that will help improve health and narrow health inequities. This said, much is already known about the potential benefits and how they are realised, and public appreciation for parks and other NBIs has such long–standing support that many generations of urban residents have already been able to benefit from their availability.

In this study, we used an expert elicitation process to compile a list of the nature–based health interventions that have received some research attention. This process is not without its limitations. Most notably, some interventions may have been overlooked, and the list was subject to a consensus on grouping and categorisation that others may have done in a different way. Furthermore, this study has thus not systematically addressed issues of intervention efficacy, effectiveness, and efficiency. While systematic analyses of efficacy and efficiency are as yet not possible for many intervention types because of a high level of variation in the methods used, outcomes measured within the literature to date (but see, e.g., [51]), and co–benefits realised by indirect means (e.g., parks along rivers may support nature experiences and also protect homes from flooding), such evaluations will be important avenues for future research. Finally, it bears mentioning that the recognition of the possibilities with nature–based interventions is engendering considerable innovation, as with the development of therapeutic gardens for new client groups (e.g., war veterans [196]) and the use of nature experience to support the acquisition of mindfulness meditation techniques [197,198]. 

## 5. Conclusions

We have identified a suite of NBIs that can be used to improve population health and wellbeing, and to address specific physical, mental and social health issues. The identified interventions broadly fall into two categories: those that change the environment, and those that change behaviours. The selection of an intervention will require the consideration of a range of factors, including cost, likely benefit, accessibility (including availability and social acceptability) and the capacity of the organisation to deliver it. Most importantly, however, the needs of the community or the individual and the goals of the intervention must be considered. To integrate nature–based health interventions into public health and planning policy, strong evidence for their effectiveness is important, and thus evaluation should be carefully built into new interventions. 

## Figures and Tables

**Table 1:** Nature–based health and wellbeing treatment (T) or prevention (P) interventions that change environments.

Intervention	Description	T/P	Intervention Goals, and Intended Health or Wellbeing Outcome	Target Beneficiaries	Example References
**1. Provision of gardens in hospitals or residential care homes (sometimes referred to as healing gardens).**	The provision of gardens that can either be viewed from hospital rooms or accessed by patients and families (can include green walls).	T	Reduce pain and stress, potentially leading to improved healing time and mental health, quality of life, wellbeing, reduced agitation for patients with dementia.	Hospital or residential care patients, their families and friends, staff. Can have targeted groups in some circumstances, e.g., hospitals for patients living with dementia.	[36,37,38,39,40,41,42,43,44]
**2. Provision of nature within rooms in healing environments.**	The provision of nature that can be viewed or experienced from a person’s room and/or in shared areas (e.g., the view from a window, or indoor plants, flowers, garden, green walls).	T	Reduce pain and stress, potentially leading to improved healing time and mental health, social contacts, quality of life, wellbeing.	Hospital or residential care patients, their families and friends, staff.	[40,41,42,45,46,47,48,49,50,51]
**3. Indoor plants in workplaces or other non–healing indoor environments such as shopping centres.**	Organisations shape policies and make provisions for indoor plants.	P	Enhance creativity, improve productivity, reduce absenteeism at work, improve mental wellbeing, improve air quality.	Those using indoor environments.	[52,53,54]
**4. Increased provision of public urban parks and gardens.**	Additional new parks are provided.	P	Parks are provided to encourage outdoor leisure, engagement with nature, increase neighbourhood walkability and physical activity, with some of the cited health benefits including the physical benefits from exercise, enhanced social cohesion, mental wellbeing and quality of life outcomes.	Neighborhoods or entire towns.	[18,26,34,55,56,57,58]
**5. Improvement of urban public parks and gardens.**	Improvement could include: (i) better public access to existing parks, including public transport provision and accessibility for those with disabilities, and improved equality in access across socioeconomic gradients; (ii) better street lighting and passive surveillance to reduce fear of crime; (iii) traffic reduction measures to reduce pollution and noise; (iv) enhancement of biodiversity within parks.	P	Improvement of parks to enhance community engagement with under–utilised parks and improve biodiversity to enhance the restorative benefits received. Some of the cited health benefits of parks include exercise, enhanced social cohesion and mental wellbeing and quality of life outcomes.	Neighborhoods or entire towns.	[59,60,61,62,63,64]
**6. Provision of walking or bike paths, or other shared use paths/trails.**	Areas designed specifically for walking or biking. Includes paths through parks or natural areas that facilitate active travel.	P	Provide a facility that encourages physical activity, delivers the associated benefits, and improves general wellbeing.	General population in an area.	[65,66,67,68,69,70,71,72,73,74,75]
**7. Streetscape enhancement/green corridors along streets.**	Councils plant vegetation along streets and support the efforts of residents to plant vegetation in their private or community gardens (includes both native and non–native species).	P	Enhance the environment for attention restoration, in part by improving the view from people’s homes. Indirect health benefits include better air quality, reduced heat island effects.	Neighborhoods or entire towns.	[76,77,78]
**8. Community gardens/allotments.**	Gardens in accessible locations for community members to encourage engagement in growing one’s own food and to provide food education involving fruit and vegetables.	P	Improve nutrition, social connections and psychological benefits (e.g., confidence, psychological restoration).	Neighbourhoods or entire towns, sometimes with specific intended beneficiaries (e.g., age groups).	[79,80,81,82,83,84,85,86]
**9. Greening childcare or school grounds.**	Increase amount and quality of natural elements, including around classrooms and play areas.	P	Increase physical activity, increase imaginative play, development of positive relationships, place of learning, attention restoration, overall improvement in health.	Children using the facility.	[87,88,89,90]
**10. Outdoor gym equipment.**	Provide alternative exercise facilities, specifically outdoor versions of traditional gym equipment.	P	Encourage physical activity and promote the associated benefits and increased wellbeing in those reluctant to use traditional gyms or more motivated by being outdoors.	Neighbourhoods or entire towns, those reluctant to go to indoor gyms.	[91,92]
**11. Provision of accessible natural environments.**	Location and spatial planning of accessible natural environments, with paths. Infrastructure created or improved in local woodlands, and a programme of social engagement.	P	Increase use of natural environments for health, recreation, leisure, etc. to facilitate health and wellbeing outcomes such as reduced stress, improvements in mood.	Local residents and wider populations.	[93,94,95,96]

**Table 2:** Nature–based health and wellbeing treatment (T) or prevention (P) interventions that aim to change the behaviour in individuals or groups with specific physical, mental or social health and wellbeing issues. ADHD: attention–deficit/hyperactivity disorder.

Intervention	Description	T/P	Intervention Goals (i.e. Health Outcome)	Target Beneficiaries	Barriers to Implementation, Unintended Negative Impacts	Example References
**12. Green/nature/park/garden prescriptions.**	Doctors (or other professionals) ‘prescribe’ or refer patients/clients to outdoor activities (often walks).	P/T	Increase exercise and the associated benefits, stress reduction, reduce blood pressure, improve healing times, reduce depression, increase resilience and other mental health benefits. Some are targeted towards children for purposes such as prevention or treatment of obesity, cancer and diabetes. Some also target quality of life, wellbeing and social support.	Individual patients or groups with a range of conditions.	*Individual-level barriers:* Geographic accessibility and availability of facilities (e.g., green spaces), affordability of the activity, social acceptability, physical and cognitive capability of individuals, perceived issues such as danger. *Organisation-level barriers:* Acceptability by and lack of knowledge of medical professionals, difficulty in changing behaviours of medical professionals. *Potential unintended impacts:* Could present risks for people with some conditions.	[31,32,97,98,99,100,101,102,103,104]
**13. Care–farming or farm therapy, including horticulture and animal–assisted therapy.**	Therapeutic use of commercial farms and agricultural landscapes as a base for promoting mental and physical health, through normal farming activity or horticulture.	T	Mental health promotion and to reduce distress in people with dementia. Reduce social isolation.	Youth at risk; youth with special needs (e.g., autism); cancer survivors; mental disorders; people with lost functionality; people recovering from serious illness.	Not assessed in this study.	[83,105,106,107,108,109,110,111,112,113,114]
**14. Residential retreats.**	Multi–modal therapies delivered in a removed natural setting.	T	Holistic wellbeing: physical, but primarily psychological (coping), social, spiritual.	Patients with chronic conditions such as cancer or cardiovascular disease.	Not assessed in this study.	[115]
**15. Wilderness therapy.**	Structured nature–based activities and programmes in ‘wilder’ environments for ‘at risk’ groups or those recuperating or in recovery	P / T	Address social and psychological issues through a range of pathways, including by facilitating positive human–nature interactions, building self–esteem and fostering social connections.	People with severe mental health issues; youth at risk of involvement in crime; individuals who are imprisoned or on probation from crime; ex–offenders; victims of crime; children with ADHD; those living with or recovering from a range of mental and physical conditions; people with post–traumatic stress disorder.	*Individual-level barriers:* Geographic accessibility and availability of facilities (e.g., green spaces), affordability of the activity, social acceptability, some people may not appreciate the group context, physical ability, time (several days often required). *Organisational level barriers:* Poor system support, lack of financial resources to support the activities. *Potential unintended impacts:* Mental distress and physical injury in poorly managed activities, poor follow–on care. Often offered as a once–in–a–lifetime developmental boost, and they may be required more often.	[111,116,117,118,119,120,121,122,123,124,125]
**16. Wilderness programmes.**	Programmes designed to challenge participants in natural environments.	P	Personal growth, social skills.	Often youth, but also targeting any interested people and groups.	Not assessed in this study.	[126,127]
**17. Ecotherapy.**	Treatment modalities that include the natural world in relationships of mutual healing and growth, and as such are a form of applied ecopsychology.	T	Positive effects on psychological wellbeing, fitness and self–reported health.	People with symptoms of stress, or other mental health and wellbeing issues.	Not assessed in this study.	[128,129,130,131]
**18. Pet therapy, or pet–assisted therapy.**	Use of pets, especially in hospitals to benefit patients.	T	Psychological wellbeing; social wellbeing.	Hospital inpatients; other vulnerable groups.	Not assessed in this study.	[132,133,134]
**19. Forest bathing.**	Practice of spending time in forest settings, often with emphasis on attention to breathing and other meditative techniques	P / T	Improved physical and mental wellbeing.	People referred to the program or voluntary participation.	Not assessed in this study.	[95,96,100,135,136]
**20. Green gyms or environmental volunteering.**	Active work in an outdoor environment, often with a focused conservation outcome.	P/T	Provide diverse benefits including physical activity, mental wellbeing, social connection/(re)integration.	People referred to the program or voluntary participation.	*Individual-level barriers:* Geographic accessibility (including transport) and availability of facilities (e.g., green spaces), affordability of the activity, social acceptability, availability of the programmes. *Organisation-level barriers:* Lack of financial resources, acceptability by and lack of knowledge of health professionals, difficulty in changing behaviours of health professionals. *Potential unintended impacts:* Chance of injuries and risk of other negative impacts of nature (e.g., insect bites, allergic responses), conflict in management of green spaces. Limited knowledge by host organisations of how to supervise people with physical or mental impairment.	[137,138,139,140,141,142,143,144]
**21. Outdoor exercise groups.**	Groups with the specific aim of exercising in nature (most commonly walking) for health benefits.	P/T	Improve physical, psychological, social and spiritual wellbeing, including better cardio–vascular health, psychological wellbeing.	Local interested residents, or people referred to the program with a specific health condition, or voluntary participation.	*Individual-level barriers:* Geographic accessibility and availability of facilities (e.g., green spaces), affordability of the activity, social acceptability, concerns about, e.g., getting muddy or other issues, unfamiliarity with using non–urban environments, personality (e.g., introverts may elect out), mobility issues. *Organisational-*level barriers: Lack of financial resources or certainty, communication preferences for older individuals (e.g., social media). *Potential unintended impacts:* Chance of physical injury, group setting may engender negative feelings and interactions.	[72,128,145,146,147,148,149,150]
**22. Nature play/wild play.**	Structured programmes designed to facilitate children’s play in natural environments.	P	Enhance child health and development through provision of social programmes and physical environments that promote varied play opportunities, improved attention and learning, physical activity, mental health.	Children (general).	Not assessed in this study.	[151,152,153,154,155,156,157]
**23. Forest Schools/outdoor classrooms/learning environment.**	Programme of education in the outdoors (rather than about the outdoors). Typically children spend a period of their schooling (ranging from a couple of hours a week to all their time) undertaking outdoor activities. Forest school is both a pedagogy and a physical entity, with the use often being interchanged.	P	Provide alternative (and sometimes improved) learning environment, increase physical activity and the associated benefits.	Typically children, but has been used with adults and people with special needs.	Not assessed in this study.	[158,159,160,161]
**24. Children’s kitchen gardens.**	Gardens in schools and kindergartens to encourage engagement in growing one’s own food and to increase access to fruit and vegetables	P	Improve nutrition, social connections and psychological benefits (e.g., confidence, team work skills), physical activity, educational outcomes, school–based quality of life.	Children in childcare, nurseries and schools.	Not assessed in this study.	[162,163,164,165,166,167,168,169,170,171]
**25. Outdoor education schemes.**	Schemes designed to introduce children/adults to nature with the purpose of altering their knowledge about, attitudes toward and contact with nature.	P	Increase confidence to use natural environments for physical activity and recreation and promote the health and wellbeing benefits associated with this and increased nature exposure.	Largely children, but also aimed at adults from vulnerable groups (e.g., rehabilitation) and others.	Not assessed in this study.	[172]
**26. Promotion and facilitation campaigns.**	Promotional campaigns (e.g., via media) to highlight and encourage engagement with natural environments and potential health benefits.	P	Increase awareness, engagement, use and experience of natural environments.	General population, but often targeted at specific groups such as different age groups.	Not assessed in this study.	[128,173]
**27. Blue gym.**	Water– or shoreline–based activities.	P	Improve mental wellbeing.	General population.	Not assessed in this study.	[174]
